# Walking with a Posterior Cruciate Ligament Injury: A Musculoskeletal Model Study

**DOI:** 10.3390/bioengineering10101178

**Published:** 2023-10-11

**Authors:** Lucia Donno, Alessandro Galluzzo, Valerio Pascale, Valerio Sansone, Carlo Albino Frigo

**Affiliations:** 1 Movement Biomechanics and Motor Control Lab, Department of Electronics, Information and Bioengineering, Politecnico di Milano, I-20133 Milan, Italy; carlo.frigo@polimi.it; 2 IRCCS Istituto Ortopedico Galeazzi, I-20161 Milan, Italy; alegalluzzo@gmail.com (A.G.); valerio.pascale@unimi.it (V.P.); valerio.sansone@unimi.it (V.S.); 3 Residency Program in Orthopaedics and Traumatology, University of Milan, I-20122 Milan, Italy; 4 Department of Biomedical Sciences for Health, University of Milan, I-20122 Milan, Italy; 5 Department of Biomedical, Surgical and Dental Sciences, University of Milan, I-20122 Milan, Italy

**Keywords:** musculoskeletal modeling, PCL injury, knee joint biomechanics, knee ligaments

## Abstract

The understanding of the changes induced in the knee’s kinematics by a Posterior Cruciate Ligament (PCL) injury is still rather incomplete. This computational study aimed to analyze how the internal loads are redistributed among the remaining ligaments when the PCL is lesioned at different degrees and to understand if there is a possibility to compensate for a PCL lesion by changing the hamstring’s contraction in the second half of the swing phase. A musculoskeletal model of the knee joint was used for simulating a progressive PCL injury by gradually reducing the ligament stiffness. Then, in the model with a PCL residual stiffness at 15%, further dynamic simulations of walking were performed by progressively reducing the hamstring’s force. In each condition, the ligaments tension, contact force and knee kinematics were analyzed. In the simulated PCL-injured knee, the Medial Collateral Ligament (MCL) became the main passive stabilizer of the tibial posterior translation, with synergistic recruitment of the Lateral Collateral Ligament. This resulted in an enhancement of the tibial–femoral contact force with respect to the intact knee. The reduction in the hamstring’s force limited the tibial posterior sliding and, consequently, the tension of the ligaments compensating for PCL injury decreased, as did the tibiofemoral contact force. This study does not pretend to represent any specific population, since our musculoskeletal model represents a single subject. However, the implemented model could allow the non-invasive estimation of load redistribution in cases of PCL injury. Understanding the changes in the knee joint biomechanics could help clinicians to restore patients’ joint stability and prevent joint degeneration.

## 1. Introduction

The knee joint is a remarkably complex system from both the anatomical and functional points of view, with very specific biomechanical requirements. In the sagittal plane, it allows a wide range of flexion/extension in the presence of major loads resulting from body weight and inertia forces. In the coronal plane, it provides a high degree of stability throughout the full range of motion, and concerning the transverse plane it allows internal/external rotation when the knee is flexed. All these functional properties result from the specific morphology of the joint contact surfaces and the presence of ligaments.

In recent decades, plenty of literature was published regarding the role of cruciate ligaments and the effects of ligament deficiency in anterior–posterior stability, both in the native and the prosthetic knee. Most of the scientific attention was devoted to the Anterior Cruciate Ligament (ACL), probably due to the particular epidemiology of knee joint injuries, showing that the ACL is affected significantly more frequently than other fibrocartilaginous and ligamentous structures [1]. In contrast, the Posterior Cruciate Ligament (PCL) has been less investigated, despite being a pivotal structure within proper knee biomechanics. This ligament is an intra-articular extra-synovial cord of dense connective tissue, consisting of two distinct bundles, i.e., anterolateral and posteromedial bundles [2]. It plays a crucial role in resisting posterior tibial translation relative to the femur during knee motion, and recent literature enlightens the importance of this structure in maintaining the rotational stability of the knee, especially between 90° and 120° of flexion [3,4,5,6].

The role of the PCL is crucial also in the choice between cruciate-retaining and posterior-stabilized prosthetic implants [7]. If the cruciate-retaining implants could better preserve the physiological kinematics of the knee [8,9], their application becomes challenging in cases of PCL laxity due to the problematic balancing of the ligaments [10]. However, in the case of cruciate-retaining implants, recent findings showed that the mobile bearing design could ensure the stability of the knee joint even in case of PCL deficiency [11].

Although these findings are fairly well accepted in the scientific community, the understanding of the changes induced in the knee kinematics by a PCL injury is still rather incomplete. The large cross-sectional area of this ligament would lead us infer that it must bear significant loads, but in practice the functional impairments induced by an isolated injury of this ligament are essentially limited. In fact, many surgeons prefer to treat isolated injuries of the PCL conservatively. The PCL retention could influence not only the knee kinematics and stability, but also the shear forces on the tibial contact surface and the proprioception [12,13,14]. The surgical treatment of ligament reconstruction is therefore reserved for rare cases of significant and persistent functional limitations. The relative paucity of symptoms suggests that there are intrinsic forms of compensation that the joint can exploit when a ligament injury occurs. One of these could be the greater healing capacity of the PCL compared to the ACL as reported in the literature, probably due to a better synovial coverage and perfusion [15]. Patients with isolated PCL tears may have few functional problems in the short-medium term, but there is growing literature enlightening the detrimental effects of PCL deficiency in terms of acute or degenerative injury of the remaining joint structures and development of early arthrosis [16,17,18].

In general, the analysis of weightbearing activities (i.e., walking and climbing stairs) is limited in the literature. Specifically, for the task of walking, the effects of PCL lesion remain unclear and are worthy of further investigation [19]. Understanding the changes in the knee joint biomechanics could help clinicians to restore patients’ joint stability and prevent joint degeneration. The inherent complexity of the knee joint and the close interdependence between the various elements makes the study of individual anatomical structures and their pathophysiology particularly difficult, and this applies specifically to PCL. In addition, this ligament injury is relatively infrequent, and the isolated rupture is rare. In an observational study of 85 PCL injuries, Fanelli G.C. et al. [20] reported that isolated PCL ruptures were no more than 3.5% (3 of 85), whereas 95.5% (82 of 85) of PCL injuries occurred in combination with other ligament lesions. This indicates the difficulty of analyzing the effect of this particular lesion on the knee biomechanics while also having regard to the dynamic interaction with other articular structures.

Computational musculoskeletal models effectively overcome this difficulty by enabling the researcher to observe the effect of a single-element modification on the overall joint motion and biomechanics. This is one of the main reasons why research on musculoskeletal models has grown exponentially (the number of scientific publications has increased by nearly 1500% in the last 20 years). Musculoskeletal models have proven to be fundamental tools for improving our understanding of the musculoskeletal system and related pathological conditions [21,22]. For example, in a previous study [23], the effects on knee joint kinematics and internal load distribution produced by removal of the ACL were simulated with reference to the walking cycle. In that study it was also possible to investigate the effects of different muscle contractions, the compensatory role of quadriceps and hamstrings in particular.

Kang et al. [24] simulated a PCL deficiency in a subject-specific knee model through a force-dependent kinematics method under gait and squat loading conditions. The cited authors found out that the PCL deficiency affects the contact forces of the tibial–femoral and patellar–femoral joints less during the gait cycle with respect to the squat loading condition. However, this study did not focus on the effects of PCL injury on the knee kinematics under dynamic conditions, but only on passive flexion and posterior drawer tests. Moreover, to our best knowledge, no other studies investigated on the impact of different degrees of PCL lesion on knee kinematics during walking.

In the present study, several dynamic simulations of the gait cycle were performed aimed at answering to two main questions: (i) how are the internal loads redistributed among the remaining joint ligaments when the PCL is lesioned at different degrees? (ii) is there a possibility to compensate for a PCL lesion by changing the hamstring’s contraction in a particular phase of the stride cycle?

## 2. Materials and Methods

### 2.1. Modeling Approach

**Figure 1:**
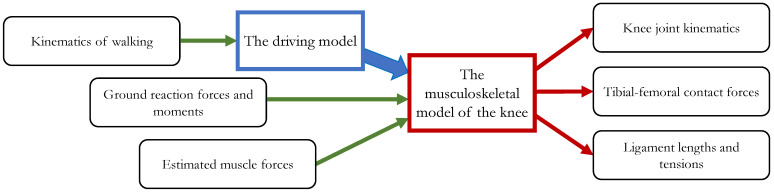
Schematic overview of the numerical research procedure.

The dynamic simulations of the gait cycle were performed by means of a previously developed three-dimensional musculoskeletal model implemented on the SimWise-4D platform (Design Simulation Technologies, DST, Canton, MI, USA) [25]. This software performs both the forward and the inverse dynamics of complex articulated systems and solves the mechanical interaction between body surfaces. Figure 1 represents a schematic overview of the numerical procedure. The integration algorithm used to solve the dynamic equilibrium equations was based on the Kutta–Merson process. The following parameters were adopted: integration step 0.02 s, configuration tolerance 0.01 mm and 0.1°.

**Figure 2:**
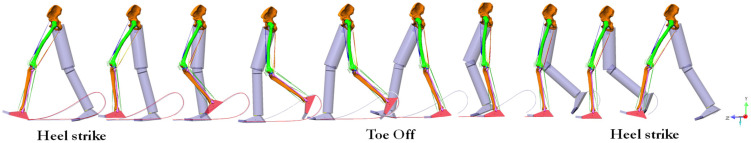
The walking model simulating the gait cycle (from right to left). Gait cycles events (heel strikes and toe off) are referred to the left lower limb, including the detailed knee joint model.

The implemented model included a “driving model”, composed of trunk, pelvis, thighs, shanks and feet, and a detailed “knee joint model” attached to it. The anatomical segments in the driving model, represented by rigid bodies, were linked together by rotational actuators (motors), by means of which it was possible to impose the desired kinematics, in our case the walking cycle (Figure 2). The pelvis was linked to the trunk by three rotational actuators controlling 3 degrees of freedom (d.o.f): anteversion/retroversion, frontal plane tilt and horizontal rotation. Three other motors were used to control the relative movement between the thigh and the pelvis (hip flexion/extension, adduction/abduction, internal/external rotation). Similar actuators controlled the 2 d.o.f between the shank and the thigh (knee flexion/extension and internal/external rotation) and the 2 d.o.f between the foot and the shank (ankle plantar/dorsiflexion and pronation/supination).

All these motors received as input the kinematic data from our repository, referring to the average of 14 strides recorded on 5 healthy male subjects aged between 24 and 36 years, walking barefoot at their self-selected speed. These data (trajectory of a reference point on the trunk and joint angles corresponding to the d.o.f of the model) were obtained by applying the SAFLo (Servizio di Analisi della Funzionalità Locomotoria) marker set protocol [26] and were normalized in time so as to obtain an ideal walking cycle lasting 1 s. To ensure that the results were obtained at steady state condition, two subsequent walking cycles were simulated, and only the second one was analyzed. Anthropometric tables [27] provided masses of body segments corresponding to a healthy subject with a height of 1.72 m and 70 kg of body mass.

The knee joint model was composed of the digital models of the femur and tibia, obtained by digital segmentation of MRI images (Amira 5.3.3, Visage Imaging, Inc., San Diego, CA, USA) of a 42 year old Caucasian male who was 1.72 m tall and had 70 kg of body mass. The two femoral condyles were in contact with the tibial plateaus and could slide along the contact surfaces. In order to obtain a proper sliding between the contact surfaces, the portions of the distal femur and of the proximal tibia were separated from the rest of the bones and their contact surfaces were smoothed by using a commercial software (Meshmixer 3.5.47, Autodesk, San Rafael, CA, USA). Then, for the purpose of reducing the simulation time, the two portions were imported again in the software SimWise4D and connected to the respective bones by rigid constraints; thereby, the algorithms analyzing the surface interaction had to work only on these small portions of the bones. Specifically, a friction coefficient equal to 0.01 between the contact surfaces of the tibia and the femur was set [28] and their interaction was modeled as an inelastic collision. The two bones were connected by springs representing the knee joint ligaments. The knee joint model was introduced in the driving model by rigidly connecting the femur to the thigh. A specially designed device called a “Grood&Suntay mechanism (G&S)” (the name comes from the convention adopted for the definition of the knee joint d.o.f. [29]) was used to impose the flexion/extension movement of the knee while keeping the other 5 d.o.f. unconstrained. Thus, the movement of the tibia relative to the femur was determined by the geometrical interaction between the surfaces of femoral condyles and tibial plateau, the tension produced by the ligaments, and all the forces acting on this structure (muscle forces, ground reaction forces, weight and inertial forces of shank and foot). The relative movement between the tibia and femur was measured by the G&S mechanism in terms of adduction/abduction, internal/external rotation and proximal/distal, medial/lateral and anterior/posterior tibial displacements. As for the muscle forces, an estimate was obtained by implementing an optimization procedure for the minimization of the maximum force (Min/Max criterion [30]) in relation to the muscle’s physiological cross-sectional area. The muscle lever arms were obtained by analyzing the moment produced by each muscle when it was activated with a predefined muscle force. Each muscle acting on the knee joint was modeled as a force actuator producing the respective estimated concentric force. The quadriceps group was represented by four actuators corresponding to the Vastus Lateralis, Vastus Medialis, Vastus Intermedius and Rectus Femoris. The hamstrings group was considered to be composed of the Semitendinosus, Semimembranosus, Biceps Femoris long head and Biceps Femoris short head. The Gastrocnemius Lateralis and Gastrocnemius Medialis were also implemented in the model, acting as knee flexors.

The ground reaction forces (anterior–posterior, medial–lateral and vertical components) measured during walking were applied to the knee model. Through an inverse dynamics analysis, the inertia forces and moments associated with the movement of the shank and foot were computed.

**Figure 3:**
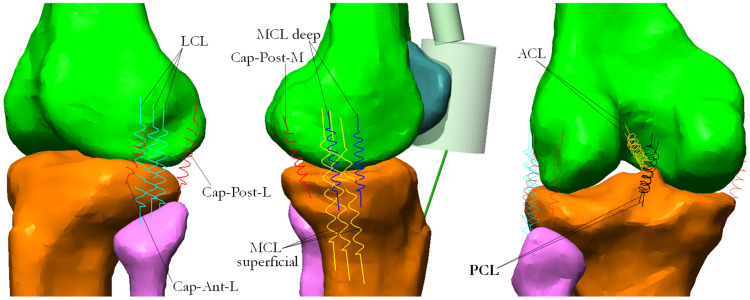
Springs with non-linear characteristics represented the ligaments connecting femur and tibia. On the left, Lateral Collateral Ligament and anterior and posterior lateral bundles of fibrous capsule. In the middle, the extensor mechanism, superficial and deep bundles of Medial Collateral Ligament and medial portion of fibrous capsule. On the right, Anterior and Posterior Cruciate Ligaments.

A detailed ligament structure was implemented in the knee model. Each ligamentous fascicle was represented by a straight spring with non-linear behavior (viscoelastic element). Hence, for each ligament, several springs were included to represent the different fascicles that may be tensioned in different working conditions (Figure 3). The Anterior (ACL) and Posterior (PCL) Cruciate Ligaments were subdivided into two springs each. Three springs representing the anterior, intermediate and posterior fascicles were used both to model the Lateral Collateral Ligament (LCL) and the superficial Medial Collateral Ligament (MCL). The deep fascicles of the Medial Collateral Ligament (MCL deep) were modeled by two additional springs. The fibrous capsule was also included in the knee model and was represented by its antero-lateral (Cap-Ant-L), posterior-lateral (Cap-Post-L) and posterior-medial (Cap-Post-M) bundles. The springs representing the ligaments were attached to specific points identified with the support of many publications [31,32,33,34,35] and a joint physiology text [36]. Each spring was characterized by a quadratic rise of the force up to a predefined strain limit (assumed equal to 0.03) and a linear behavior for larger deformations, in accordance to [31,37,38]. As regards the stiffness parameter associated to the non-linear springs, the values reported by [33] were adopted. Specifically, for those ligaments appearing as single units in the cited study but split in more bundles in our model, the stiffness of each elastic element was obtained by dividing the reported value by the number of bundles constituting the ligament.

As for the extensor mechanism, to reduce the computational time the rotula was reproduced by a cylinder sliding on the femoral trochlea and having the lower extremity attached to the tibial tuberosity by a rope representing the patellar tendon (Figure 3). Its upper extremity was linked by a series of smaller cylinders reproducing the quadriceps tendon, which in turn were attached to the muscle actuators. The cylinders were connected to each other by a spherical joint. The cylinder representing the rotula was put in collision mode with the femur.

Interested readers can find an exhaustive description of the implemented model in our previous publication [25].

### 2.2. Model Validation

The performance of our intact knee model was assessed in detail in our previous publication [25] by looking at three major determinants of the knee function during the whole gait cycle. First of all, we considered the knee kinematics, since in our model only the flexion/extension movement was imposed and the remaining 5 d.o.f were completely free. A forward displacement of the tibia with respect to the femur was observed during each of the two knee flexion phases, the first during the stance phase and the second associated with the swing phase. This was consistent with the consolidated knowledge about the association of sliding and rotation in the tibial–femoral movement. Secondly, in the mentioned study, we observed the presence of the well-known “screw home mechanism”: the tibia experienced an external rotation when approaching the full extension and, conversely, showed an internal rotation during the initial flexion of the knee.

Thirdly, we analyzed the tibial–femoral contact forces. In our model, the values obtained were larger than the force measured by instrumented prostheses [39,40,41], but the main features [42] were well reproduced: they exhibited two peaks during the stance phase (second peak higher than the first one) and a third peak at the end of the swing phase, ensuring the joint stability. To make a quantitative assessment, we made a comparison between our data and those provided as a reference for the Grand Challenge competition [41]: after downscaling our results, the RMS difference with respect to the reference data was 318 N. This result shows that the predicted time-course reproduces the force measured in vivo well. As for the amplitude, it must be considered that our input data for the model are probably different from those of the subjects analyzed in the reference study (operated by knee arthroplasty) in terms of anthropometric measures and walking speed. In our musculoskeletal model, the first peak reaches 3.5 BW and the second peak is equal to 4.8 BW. These values are similar to the results obtained by Hu et al. [43]: 4 BW and 4.7 BW for the first and the second peaks, respectively. Moreover, the value we predicted for the first peak lies within the range of many studies published in the literature: Winby et al. [44] reported 3.2–4.9 BW, Meireles et al. [45] obtained 3.3–4.8 BW, and in Morrison et al. [46] 2.1–4.0 BW was found.

Thus, in terms of amplitude, the tibial–femoral contact force estimated in our intact knee model was higher than the force measured in vivo using sensorized prostheses, but in agreement with several studies that estimate the amplitude through computational models.

Further comparisons were made with data in the literature considering the typical clinical functional tests aimed at assessing the ligament’s condition. The knee model was subjected to the typical anterior–posterior drawer, varus–valgus and internal–external rotation tests performed under passive conditions and different flexion angles. The predicted stiffness and laxity of the knee joint were compared to the experimental results obtained by Markolf et al. [47] on cadaver specimens, and proved to be in relatively good agreement.

Finally, as for the muscle forces, our approach looked for the minimization of the maximum force referred to the muscular physiological cross-sectional area and our results were consistent with the well-known electromyographic patterns [48], as was the case for most of the published works [49,50,51,52,53].

### 2.3. Simulation Conditions

**Figure 4:**
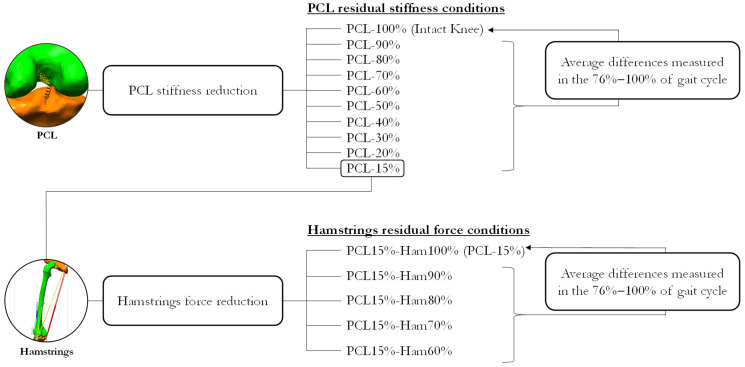
Workflow of the study.

In this study, two sets of simulations were performed to respond to two main questions (Figure 4). The first objective was to quantify the effects of different degrees of PCL injury on the knee kinematics, ligaments load redistribution and tibial–femoral contact force. Starting from the ‘IntactKnee’ model, a progressive PCL injury was simulated by reducing gradually, by 10% steps, the stiffness of the two non-linear springs representing the PCL. Thus, the conditions of PCL residual stiffness from 90% to 20% were obtained. Then, in this study, the most critical condition was obtained assuming a residual stiffness of 15% of the original one. In each of the nine simulated conditions, a dynamic simulation of the gait cycle was run and the ligament’s tension, contact force and knee kinematics in each PCL injury condition were analyzed. In particular, we quantified the changes occurring in the phase of maximum PCL recruitment, the second half of the swing phase, from 76% to 100% of the stride cycle.

The second objective of this study was to understand if the hamstrings muscle group could compensate for PCL injury. Hence, starting from the most critical condition (PCL residual stiffness being 15% of the original, named “PCL-15%”), the force produced by each hamstring’s muscle (Semitendinosus, Semimembranosus, Biceps Femoris long head and Biceps Femoris short head) was progressively reduced by 10% steps. Four new dynamic simulations of walking were performed, corresponding to a hamstring residual force of 90%, 80%, 70% and 60% of the original, having a PCL residual stiffness at 15%. Knee kinematics, ligaments load and tibial–femoral contact force were analyzed in all these conditions as before. Specifically, the changes occurring in the time window from 76% to 100% of the gait cycle were quantified in relation to the reference condition of 15% PCL stiffness and hamstring force at 100%.

## 3. Results

In a physiological knee joint (100% of PCL stiffness), the PCL expresses its maximum tension in the second half of the swing phase, between 76% and 100% of the stride cycle. To quantify the changes with respect to the intact knee, in each condition and for each variable, we calculated the average of the difference between the curve obtained in the altered condition and the curve computed for the intact knee in the time window 76% to 100% of the stride cycle.

**Figure 5:**
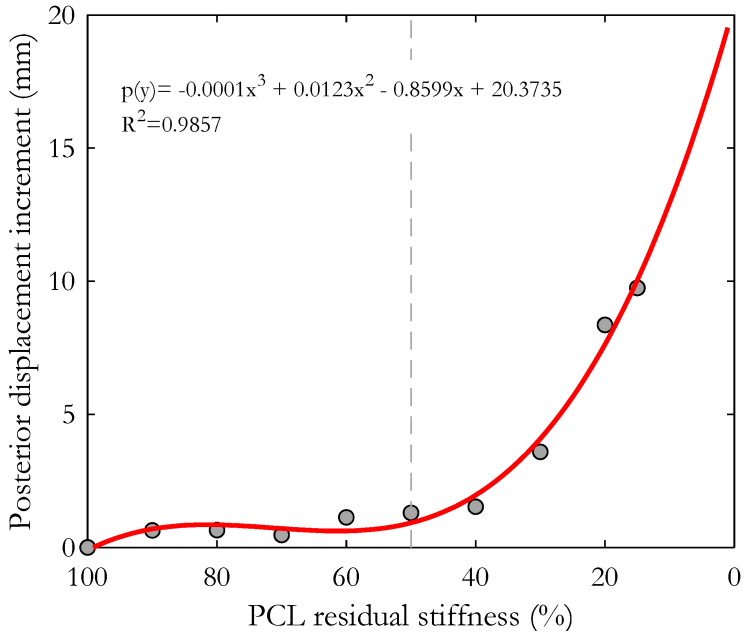
Mean increment of posterior tibial displacement calculated in the second half of the swing phase resulting from each simulated condition. In red, the polynomial regression function. Points to the right of the dashed line correspond to the most critical conditions.

Considering the posterior displacement of the tibia in relation to the femur, we realized that the most critical conditions were reached when the PCL residual stiffness was less than 50% of the physiological state. In fact, as reported in Figure 5, the posterior displacement did not increase significantly due to a decrease in stiffness up to that value.

In the following section, only the main findings obtained from the conditions of PCL residual stiffness lower than 50% will be outlined.

### 3.1. Residual PCL Stiffness from 50% to 15%

For the sake of clarity, in this section, the effects of PCL stiffness reduction on the knee kinematics, ligaments tension and tibial–femoral contact force will be outlined separately.

#### 3.1.1. Effects on Anterior–Posterior Displacement of the Tibia

**Figure 6:**
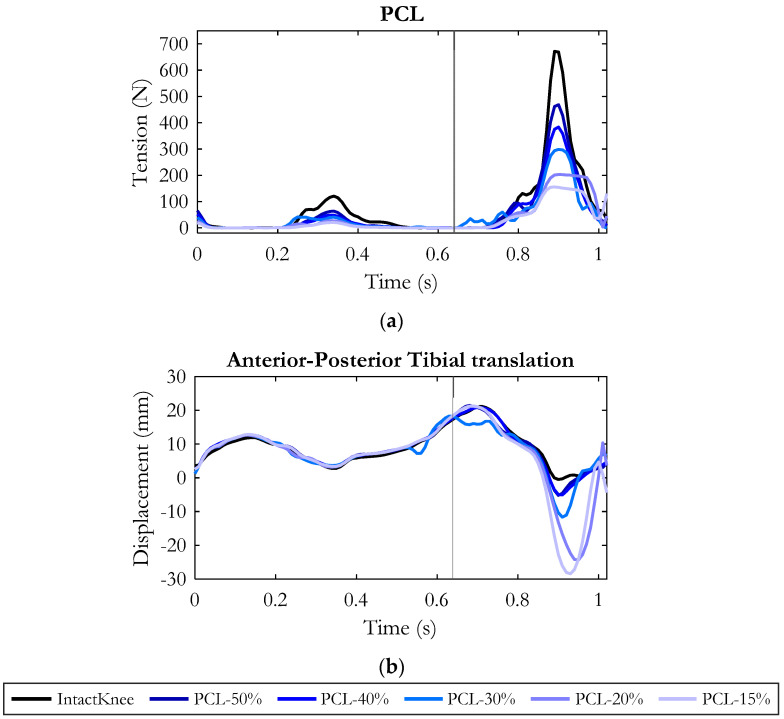
Tension of PCL in the simulated conditions (**a**). Anterior–posterior displacement of the tibia (**b**) during the gait cycle in the six simulated conditions. Values are positive for the anterior displacement. The gray vertical line refers to the toe–off event, marking the end of the stance phase and the beginning of the swing phase.

The progressive reduction in PCL stiffness consistently decreased the ligament tension all along the stride cycle, as shown in Figure 6a. The anterior–posterior displacement of the tibia during the gait cycle in the different PCL conditions is reported in Figure 6b. It appears that this variable was almost unaffected for the whole stance phase and the first half of the swing phase, while it exhibited a progressive increase in the backwards displacement in the second half of the swing phase, precisely when the PCL would express its maximum tension in a physiological knee (Figure 6a, black curve).

Specifically, in the time window considered (76% to 100% of the gait cycle), the average increase in the posterior displacement of the tibia was 1.3 mm and 1.5 mm with respect to the intact knee when the PCL residual stiffness was 50% and 40% of the healthy condition, respectively, but became 3.6 mm for a reduction in the PCL stiffness to 30% of the original one, 8 mm for a PCL stiffness of 20% and 10 mm for a PCL stiffness reduced to 15% of the original one (see also Figure 5). The peak of posterior displacement occurred at around 90% of the stride cycle, with a tendency towards lag for lower PCL stiffness.

#### 3.1.2. Effects on the Tension of the Remaining Ligaments

**Figure 7:**
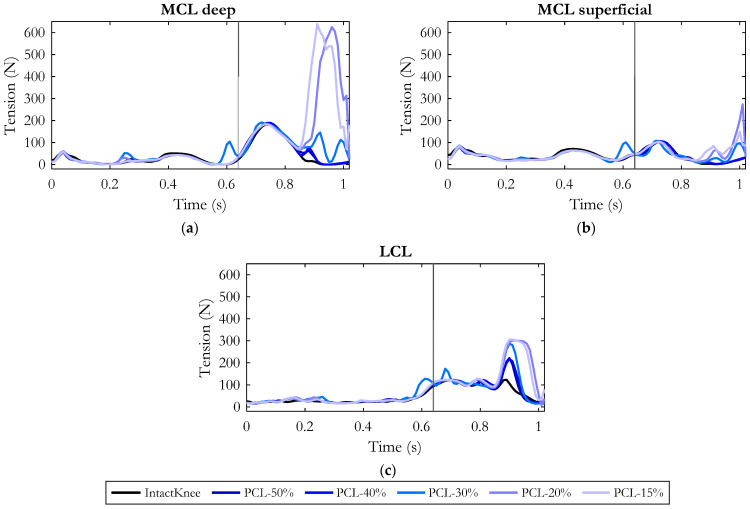
Tension of deep Medial Collateral Ligament (**a**), superficial Medial Collateral Ligament (**b**), Lateral Collateral Ligament (**c**) along the gait cycle in the six simulated conditions. The gray vertical line refers to the toe-off event, marking the end of the stance phase and the beginning of the swing phase.

Regarding the loads on the remaining ligaments, the collateral ligament structures were the most affected by the PCL injury (Figure 7). In particular, the gradual reduction in PCL stiffness resulted in a progressive increase in MCL (deep and superficial) and LCL tension specifically in the late swing phase. The average increase for a reduction in PCL stiffness to 50% and 40% compared to the intact knee condition was in both conditions about 30 N for LCL (Figure 7c). The peak of force was about 220 N and occurred at the same instant in which the injured PCL exhibited the peak of force and the tibia gained its maximum posterior displacement (90% point of the gait cycle, Figure 6).

Concerning the MCL (deep and superficial), no appreciable changes were observed until the PCL stiffness decreased to 30%. In this condition (see Figure 7a,c), a cooperation between the lateral and medial passive stabilizers was observed: the deep bundles of MCL (MCL-deep) and LCL increased their tension by approximately 50 N and 63 N on average compared to physiological conditions.

A further reduction in PCL stiffness (PCL-20%, PCL-15%) additionally demanded the recruitment of the deep and superficial layers of MCL, resulting in an average increase of 250 N and 50 N, respectively. In the same conditions, the LCL tension increased on average by 113 N.

Thanks to the combined recruitment of the passive stabilizers, at the end of the gait cycle the tibia recovered a physiological position (Figure 6b), except for the most critical condition (PCL-15%) in which the tibia remained less than 1 mm more posterior than the intact knee.

#### 3.1.3. Effects on Tibial–Femoral Contact Force

**Figure 8:**
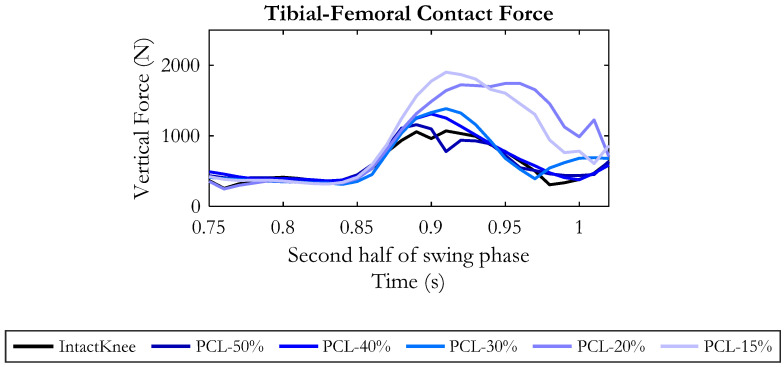
Tibial–femoral contact force in the second half of the swing phase, resulting from the six simulated conditions.

Figure 8 provides an insight into the effects of the different degrees of PCL injury on the contact force between tibia and femur in the second half of the swing phase.

Except for some small oscillations, the contact force was unaffected by the tested conditions until the 87% point of the gait cycle. Then, in the late-swing phase, the contact force increased as far as the PCL residual stiffness was reduced. With respect to the intact knee, the average force increment was 70 N for PCL-50%, 90 N for PCL-40% and 190 N for PCL-30% conditions. The average increment became dramatic for PCL-20% and PCL-15%, achieving approximately 500 N.

### 3.2. Effects of the Hamstrings on the PCL-Injured Knee (PCL-15%)

Further dynamic simulations of the gait cycle were performed keeping the PCL residual stiffness at 15% of the healthy state and gradually reducing the hamstring’s force from 100% down to 60% of the physiological condition. In this section, the main findings about the effects of hamstring force on a PCL-injured knee (PCL-15%) are reported.

#### 3.2.1. Effects on Anterior–Posterior Displacement of the Tibia

**Figure 9:**
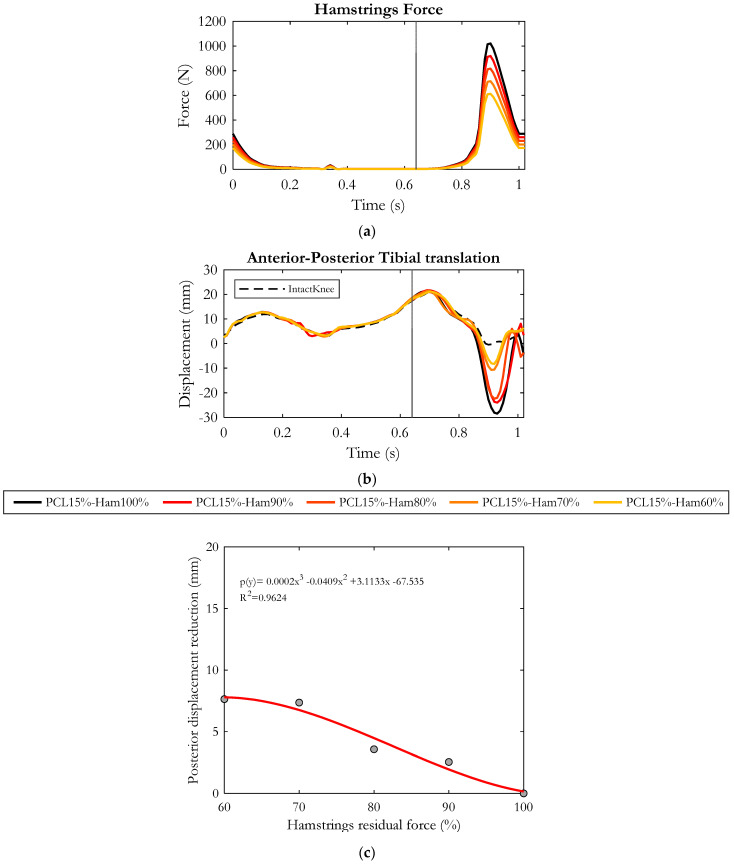
Progressive reduction of hamstring force (**a**). Anterior–posterior displacement of the tibia (**b**) during the gait cycle in the five simulated conditions. Values are positive for the anterior displacement. Dashed curve represents the intact knee condition. The gray vertical line refers to the toe–off event, marking the end of the stance phase and the beginning of the swing phase. Posterior tibial displacement average reduction with respect to “PCL15%-Ham100%” condition calculated in the second half of the swing phase (**c**), resulting from each simulated condition. In red, the polynomial regression function.

The force applied by the hamstrings to the tibia was progressively reduced, as shown in Figure 9a. Figure 9b depicts the resulting anterior–posterior displacements of the tibia. Quite obviously, the main differences were noticed in the late-swing phase, when hamstrings are activated: the gradual reduction of hamstring force resulted in a progressive decrease in the posterior tibial displacement.

Specifically, as summarized in Figure 9c, with respect to the condition in which hamstrings expressed the physiological muscular activity (“PCL15%-Ham100%”, reference condition), a force reduction to 90% lead to a decrease in the posterior displacement of the tibia by 2.5 mm on average. A further 10% drop in the hamstring’s force (“PCL15%-Ham80%”) produced a reduction of 3.5 mm on average with respect to the reference condition. When the hamstring force was reduced to 70%, the tibial posterior sliding decreased by an average of 7.3 mm; when it was reduced to 60%, the posterior displacement was reduced by 7.6 mm on average. As a consequence, the peak of backward displacement of the tibia decreased from 28 mm to 8 mm (see Figure 9b), very close to the displacement occurring in the intact knee.

#### 3.2.2. Effects on the Tension of the Remaining Ligaments

**Figure 10:**
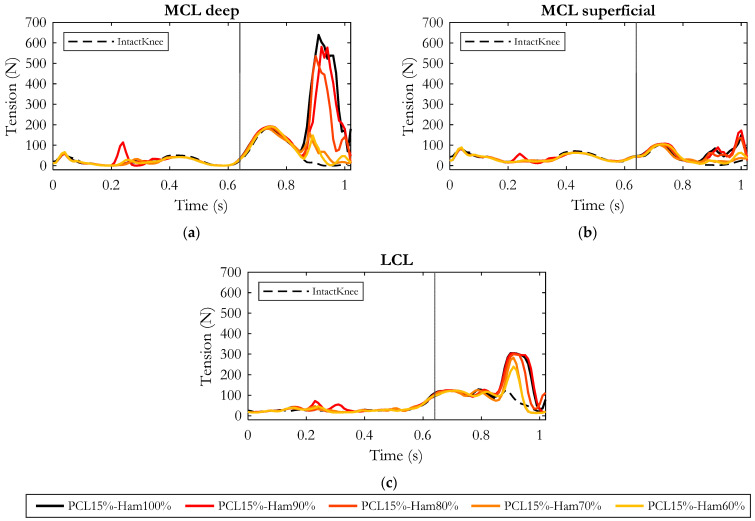
Tension of deep Medial Collateral Ligament (**a**), superficial Medial Collateral Ligament (**b**), Lateral Collateral Ligament (**c**) along the gait cycle in the five simulated conditions. Dashed curve represents the intact knee condition. The gray vertical line refers to the toe-off event, marking the end of the stance phase and the beginning of the swing phase.

Figure 10 depicts the effects of hamstring force on the tension produced by the collateral ligaments in an injured knee (residual stiffness of PCL equal to 15%). The main differences in ligament tension were observed during the hamstring’s activation phase (late-swing phase of the gait cycle). The progressive reduction of the muscular force resulted in a gradual relieving of Medial and Lateral Collateral Ligaments, as shown in the figure.

The MCL superficial gradually relaxed similarly to the other collateral ligaments, but compared to them, its variations were not remarkable. With respect to the reference condition (“PCL15%-Ham100%”), when decreasing the hamstring’s force to 90%, the deep bundles of MCL reduced their tension by 100 N on average, while in the LCL no appreciable effect was recorded. Assuming a force generated by the hamstrings equal to 80% of the physiological condition, MCL-deep experienced a further relieving with a reduction in tension of 120 N on average.

A significant tension drop (reduction of 248 N on average with respect to “PCL15%-Ham100%”) was highlighted for MCL-deep when hamstring force was decreased to 70%. In the latter case, a more noticeable effect was observed on the LCL which experienced a tension reduction of 68 N on average.

Overall, considering all the simulated conditions, the strongest effects were recorded in the case of 60% hamstring force, in which the LCL and MCL-deep were relieved by an average of 82 N and 280 N, respectively.

#### 3.2.3. Effects on Tibial–Femoral Contact Force

**Figure 11:**
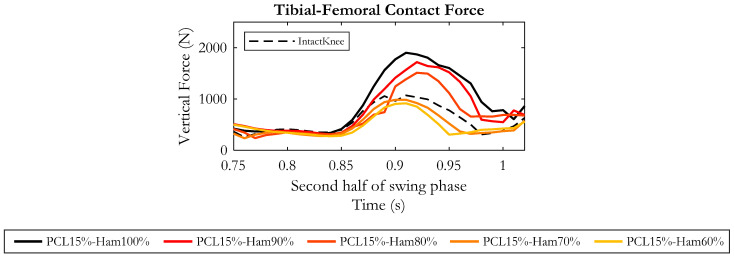
Tibial–femoral contact force in the second half of the swing phase, resulting from the five simulated conditions. Dashed curve represents the intact knee condition.

As regards the tibial–femoral contact force, in the late swing phase, a gradual decrease was recorded as the hamstring’s force was progressively reduced, as depicted in Figure 11. Compared to the “PCL15%-Ham100%” condition, in the second half of the swing phase, an average force reduction of 190 N and 280 N was recorded when the hamstring’s residual force was assumed to be 90% and 80%, respectively.

Further, an additional 10% drop in the muscular force decreased the contact force by 430 N on average, and again a decrease in the hamstring’s force to 60% lead to a mean decrease of 520 N.

Interestingly, the curves obtained in these two last conditions were below the curve of the intact knee, demonstrating that PCL injury compensated by a reduced activity of the hamstrings can reduce the total force exchanged between femur and tibia.

## 4. Discussion

In the physiological knee, the posterior traction transmitted to the tibia by the hamstrings during the second half of the swing phase is efficiently sustained by the recruitment of the PCL (as shown in the previous study [25]). Consistently, this study showed that in this time window the gradual reduction in PCL stiffness resulted in a progressive increment of the posterior tibial displacement with respect to the intact knee. Specifically, in correspondence with a PCL injury such that the residual ligament stiffness is reduced to the 50% or 40% of the healthy state, the tibial posterior displacement reached an average increase of 1.3 mm and 1.5 mm, respectively, with respect to the intact knee. The average posterior sliding of the tibia gradually augmented as the PCL residual stiffness was reduced to the most critical simulated condition (residual stiffness of 15%), in which the lesion led to an averaged increase of 10 mm in the posterior tibial displacement.

As reported by [18,54,55], one of the major complications in PCL rupture is caused by the posterior displacement of the tibia, which leads to the degeneration of both the tibial–femoral and patellofemoral joints. Consistently, the outcomes of our study highlighted that, in the late-swing phase, the contact force between femoral condyles and tibial plateaus had a mild gradual increase as the PCL residual stiffness was reduced from 50% to 30%. In the most critical conditions (PCL residual stiffness of 20% and 15%), the average increment in contact force reached up to approximately 500 N compared to the healthy condition. As reported in our findings, this effect could be related to the enhancement in the remaining ligaments’ tension (mainly in deep MCL and LCL) compensating for the PCL lesion, which resulted in increased longitudinal traction force between femur and tibia. Indeed, the contact force, as well as the tension of the remaining ligaments, was unaffected by the PCL lesion until the 80% point of the gait cycle, while considerable changes were evident from the 80% point to the end of the gait cycle, when the PCL should express its maximum force.

A typical cartilage degeneration pattern for a PCL-deficient knee presents anterior wear on the medial tibial plateau [56]. Our findings are consistent with anterior tibial cartilage wear, given the posterior translation of the tibia and the consequent anterior translation of the tibial–femoral contact point [57,58,59].

On the other hand, no significant changes were recorded on patellar–femoral contact force (not depicted in this study) and this result was in agreement with the findings of Kang et al. [24], who simulated the gait loading conditions by a subject-specific knee model. The cited authors outlined that no differences were found on patellar–femoral joint contact force between the intact and PCL-injured conditions at 0° and 60° of knee flexion during gait, while the contact force consistently increased during high flexion in the squat-loading condition.

It is worth mentioning that the patellar–femoral contact force we predicted presented two peaks in correspondence with the quadriceps contractions occurring approximately at 15% and 50% of the stride cycle [25]. Their values were 0.88 BW for the first peak and 0.74 BW for the second one. These values are within the range estimated by [60], who applied computational modeling of experimental data collected from six healthy subjects during walking. Since these contact forces occurred in correspondence with a negligible PCL tension, the change in the PCL stiffness did not affect the patellar–femoral contact force.

Actually, in our opinion, the increase in the contact force between patella and femur would occur if the backward displacement of the tibia with respect to the femur was so large that the angle of the patellar tendon with respect to the tibial plateau would change significantly. Moreover, if such a displacement occurred in a phase in which no quadriceps force is present, this effect would be not completely evident. This could explain the reason why, during the second half of the swing phase (no quadriceps activation), corresponding to the interval of the gait cycle in which the PCL would be mostly recruited under physiological conditions, no change in the patellar–femoral contact force can be appreciated. Then, for the rest of the gait cycle, the recruitment of the PCL is very limited and, consequently, as consistently outlined in this study, no appreciable changes in the kinematics, and therefore not even in the patellar–femoral force, would be expected.

Moreover, this study showed that a reduction of the contraction force of the hamstring muscles could compensate for PCL injury. Indeed, in an intact knee the PCL would be recruited during the second half of the swing phase, and at the same time the knee flexors would be activated to brake the forward movement of the shank. The required knee flexion moment is thus produced by the backward-oriented hamstring force and the forward-oriented PCL tension. In the absence of PCL, or in the case of reduced PCL stiffness, the point of attachment of the hamstrings on the tibia constitutes a sort of pivot point, so that the distal leg moves forward while the proximal tibia slides backwards, and this is depicted well in our results. Our outcomes showed that a gradual reduction of hamstring activity in a PCL-injured knee (PCL residual stiffness of 15%) resulted in a progressive reduction in the posterior tibial excursion. These findings are consistent with the well-known posterior traction effect of the hamstrings on the proximal tibia [61,62,63,64,65]. The reduction in the hamstring’s force limited the posterior sliding of the tibia and, consequently, the tension of the ligaments compensating for PCL injury (LCL and mainly deep MCL) decreased. Accordingly, thanks to the reduction in ligament tension, the tibial–femoral contact force appeared as gradually reduced.

As previously reported, a chronic anteriorization of the tibial–femoral contact point is an issue in the PCL-deficient knee, especially for the medial compartment. This could occur if the posterior sliding of the tibia is increased, and our results show that the hamstring’s force associated with PCL deficiency can be responsible for that. In this context, our results highlight the importance of targeting specific muscle groups in the rehabilitation setting during conservative treatment of PCL injury. The focus should be on promoting quadriceps recruitment, while hamstrings are best addressed with stretching and relaxation in order to avoid fostering their negative effect on tibial translation during activities of daily living [66,67].

Moreover, this study reports evidence of the compensation role of the MCL in restraining the posterior tibial translation in the case of PCL injury, and this is coherent with previous literature [68]. This observation raises awareness regarding the inherent difference between isolated PCL injury and PCL injury combined with lesions of the medial ligamentous structures. The latter condition should probably be approached with more caution, particularly during the initial phase, since one of the most important compensation structures is lacking. For example, even if at present there is limited evidence about the importance of knee bracing in the initial phase, we can assume that this indication should be stronger if the PCL lesion is combined with MCL injury.

In our model, the main limitations are the lack of cartilage and menisci between the articulating surfaces and the simplification of the patellofemoral joint. Furthermore, this study is based on a single, exemplary subject, and does not pretend to represent any specific population. Many studies showed that there is a great variability among subjects in terms of both attachment points and mechanical properties [31,32,69,70]. The realization of a subject-specific model is a challenging objective to be pursued. However, at present, the repositioning of the ligaments and changing the geometry of the joint surfaces in our model is a very delicate procedure, and this makes it difficult to adapt the model to different sizes.

However, the implemented musculoskeletal model has shown its potential in allowing the non-invasive estimation of loads on ligaments and articular surfaces and of the redistribution of loads in case of PCL injury. These aspects could hardly be evaluated in vivo.

## 5. Conclusions

The implemented musculoskeletal model appeared as a valuable tool for (i) non-invasive estimation of tension redistribution on remaining ligaments in the case of PCL injury, (ii) investigation on knee biomechanics modification in response to PCL injury and (iii) analysis of a conceivable compensatory muscular mechanism on a PCL-injured knee. The obtained results have to be considered as generic and not representative of a specific population, since the implemented musculoskeletal model refers to a single exemplary subject. However, this approach allowed us to understand and analyze the effects of a single-element modification through dynamic simulations. Moreover, it made it possible to investigate conditions hardly verifiable or reproducible in experiments, such as a gradual reduction of the hamstring’s force, as well as aspects difficult to evaluate in vivo due to ethical and technical issues, such as the ligaments’ tension or the anterior–posterior displacement of the tibia during dynamic tasks. Specifically, this computational study simulated different degrees of PCL injury and outlined their effects on the knee biomechanics: both the posterior tibial sliding and the ligaments’ load redistribution were quantified and analyzed. In the implemented model, in the case of PCL lesion, the Medial Collateral Ligament adopted the role of the main passive stabilizer of the tibial posterior translation, with a synergistic recruitment of the Lateral Collateral Ligament. However, this coping strategy resulted in an enhancement of the tibial–femoral contact force with respect to the intact knee condition and did not prevent the posterior displacement of the tibia and consequent anteriorization of the tibial–femoral contact point. This can explain the well-known conditions of tibial cartilage degeneration in a PCL-injured knee. Moreover, this study showed that reducing the hamstring activity would allow for relieving all ligaments further recruited for compensating the PCL injury and, as a consequence, limiting the contact force between the distal femur and the proximal tibia.

## Data Availability

The data presented in this study are available on request from the corresponding author.
